# Power law scaling in synchronization of brain signals depends on cognitive load

**DOI:** 10.3389/fnsys.2014.00073

**Published:** 2014-05-01

**Authors:** Jesse Tinker, Jose Luis Perez Velazquez

**Affiliations:** ^1^Neuroscience and Mental Health Programme, Brain and Behaviour Centre, Division of Neurology, The Hospital for Sick Children, TorontoON, Canada; ^2^Institute of Medical Science and Department of Paediatrics, University of Toronto, TorontoON, Canada

**Keywords:** autism, synchrony, power law, criticality, bifurcations, magnetoencephalography

## Abstract

As it has several features that optimize information processing, it has been proposed that criticality governs the dynamics of nervous system activity. Indications of such dynamics have been reported for a variety of *in vitro* and *in vivo* recordings, ranging from *in vitro* slice electrophysiology to human functional magnetic resonance imaging. However, there still remains considerable debate as to whether the brain actually operates close to criticality or in another governing state such as stochastic or oscillatory dynamics. A tool used to investigate the criticality of nervous system data is the inspection of power-law distributions. Although the findings are controversial, such power-law scaling has been found in different types of recordings. Here, we studied whether there is a power law scaling in the distribution of the phase synchronization derived from magnetoencephalographic recordings during executive function tasks performed by children with and without autism. Characterizing the brain dynamics that is different between autistic and non-autistic individuals is important in order to find differences that could either aid diagnosis or provide insights as to possible therapeutic interventions in autism. We report in this study that power law scaling in the distributions of a phase synchrony index is not very common and its frequency of occurrence is similar in the control and the autism group. In addition, power law scaling tends to diminish with increased cognitive load (difficulty or engagement in the task). There were indications of changes in the probability distribution functions for the phase synchrony that were associated with a transition from power law scaling to lack of power law (or vice versa), which suggests the presence of phenomenological bifurcations in brain dynamics associated with cognitive load. Hence, brain dynamics may fluctuate between criticality and other regimes depending upon context and behaviors.

## Introduction

Much is being discussed today about the possible critical dynamics of brain activity and its close relatives complexity and emergence. The appealing characteristics of criticality (for comprehensive introductions to the field, see Christensen and Moloney, 2005; Sornette, 2004), derived from early theoretical and computational work indicating the optimization of information processing and adaptability in general at the “edge of chaos” (Packard, 1988; Langton, 1990), fostered a tremendous interest in the application of these concepts to nervous system function (concisely reviewed in Beggs, 2007; Chialvo, 2010; Shew and Plenz, 2013), for, after all, whereas brain cells (glia and neurons) perform individually relatively simple computations, in their collective activity in the brain, cell networks achieve complex operations leading to adaptive behaviors. Critical dynamics generally show scale-invariant organization (similar fluctuations occurring at all spatio-temporal scales) which can be described by scale-invariant metrics. Of these metrics, power laws in the distribution of characteristics of the system (for instance the size of events or inter-event intervals) have been considered as typical signatures of criticality. Encouraged by early experimental observations reporting the celebrated 1/f power spectrum scaling (Pritchard, 1992; Georgelin et al., 1999), neuroscientists launched an intense investigation to study the presence of power laws in experimental recordings of all types, from *in vitro* systems to *in vivo* recordings. However, during these investigations, no consensus on the interpretation of power law scaling has emerged and many misunderstandings are currently apparent (Beggs and Timme, 2012). Most notably, while the presence of power laws is commonly thought to be associated with complexity, this association has only been formally demonstrated to occur in equilibrium statistical mechanics in systems near bifurcations. In addition, there are many means by which a system may display power laws (Mitzenmacher, 2004; Clauset et al., 2009; Marković and Gros, 2014) and some have little to do with complex dynamics.

Keeping these considerations in mind, we have assessed the possible presence of power-law scaling in a phase synchronization index of magnetoencephalographic brain recordings in children with and without autism during performance of two executive function tasks. Characterizing the difference in brain dynamics between autistic and non-autistic individuals is motivated by the potential to find differences that could either aid diagnosis or provide insights as to possible therapeutic interventions in autism. Autism and related disorders (autism spectrum disorders, ASD) are accompanied by different styles of brain information processing, reflected in some particular behavioral features of individuals with ASD. The Austrian psychiatrist Kanner (1943) described autism as “…the inability to experience wholes without full attention to the constituent parts” (even though it seems that the term autism was coined in 1911 by the Swiss psychiatrist Eugen Bleuler, who used it to describe the “withdrawal into one’s inner world”). Ideas proposed to explain the behavioral traits in ASD have mostly been on the psychological level of description, such as the weak central coherence hypothesis (Frith, 1989). With the advent of new analytical methods to scrutinize brain dynamics, especially the analysis of synchrony and “connectivity”, these ideas have been “translated” into neurophysiological notions such as disconnection amongst brain circuits. Yet, the debate still continues regarding the possible hyper or hypo-connectivity in autistic brains.^1^ What seems conceivable is that the brain coordination dynamics differs in ASD brains from others, for it is the coordinated activity of transiently formed cell assemblies that underlie cognition (von der Malsburg, 1981; Flohr, 1995; Bressler and Kelso, 2001; Kelso, 2008; Pérez Velázquez and Frantseva, 2011). Thus, studies aimed at assessing brain coordinated activity could be of relevance in the field.

Our study uses magnetoencephalographic (MEG) recordings done in two groups, children with and without ASD, performing two different executive function tasks. In our analysis, we calculated a synchronization index and studied whether the index’s empirical density function (edf) displayed power law scaling. Specifically, we looked for different expressions of power law scaling between the two groups of children and the two executive tasks. We found that power law scaling was not common and its frequency of occurrence was decreased when the cognitive load of the test was high. This difference between tasks was seen in both groups of children but little inter-group variation was observed. We discuss implications of these findings in the Discussion section.

## Materials and methods

### Participants

Data were drawn from a larger sample of children enrolled in previous studies (Pérez Velázquez et al., 2009; Teitelbaum et al., 2012). Sixteen control children (7 females) and 15 children (1 female) diagnosed with high functioning autism (Asperger syndrome) participated in the study. The children’s parents provided informed consent for the protocol approved by the Hospital for Sick Children Review Ethics Board. Age range was between 7 and 16 years. Patients met the criteria for ASD based on DSM-IV and were evaluated by the psychologists in the Autism Research Unit of the Hospital for Sick Children or were recruited from the Geneva Center for Autism and Autism Ontario. Age-matched control children had no known neurological disorders. Cognitive abilities were measured using the Wechsler Abbreviated Scale of Intelligence (WASI), as reported previously (Pérez Velázquez et al., 2009). The data analyzed in this study corresponded to 14 children (7 in each group) for the Stroop task and 25 children (12 in the ASD group) in the auditory attention task.

### Magnetoencephalographic recordings

**Figure 1:**
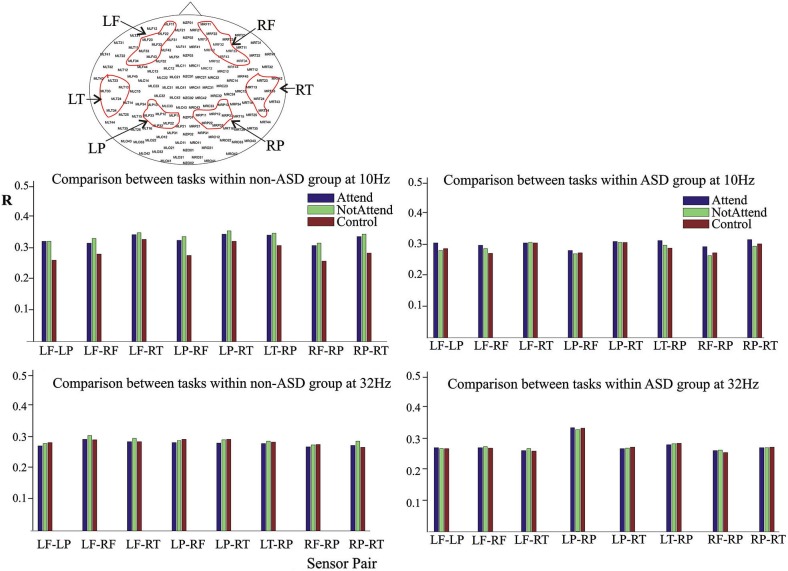
**Average magnitude of phase synchrony in the auditory attention task**. Upper head-plot depicts the MEG sensors used in the study, grouped as left frontal (LF), right frontal (RF), left parietal (LP), right parietal (RP), left temporal (LT) and right temporal (RT). The bar graphs represent the average of the phase synchrony index R (described in Section Materials and Methods) between two groups of sensors, and for each of the three conditions of the task: the baseline (“control”) condition, the attend and no-attend to the deviant tone (“Attend” and “NotAttend” respectively). For clarity, eight sensor pairs are represented (same trend was present in the rest of combinations). Note the slight increase in synchrony during task performance, more evident in the non-ASD group, at central frequency of 10 Hz, in the upper bar graphs (see details in the Results section).

MEG recordings were acquired at 625 Hz sampling rate, using a CTF Omega 151 channel whole head system (CTF Systems Inc., Port Coquitlam, Canada), as previously described (Pérez Velázquez et al., 2009). Head movement was tracked by measuring the position of three head coils every 30 ms, located at the nasion, left and right ear, and movements less than 5 mm were considered acceptable. Sensors used in the analyses are depicted in Figure 1, and were located over the following cortical areas: left and right frontal (LF, RF), left and right parietal (LP, RP), and left and right temporal (LT, RT). We chose these cortical areas as they are associated with executive functions and relatively mutually distant in space.

### Executive function tasks

#### Stroop color-word test

The color Stroop interference paradigm is a commonly used test of inhibition (Stroop, 1935), in which the participant names the colors of the ink in which words are written. It consists of a list of color words written in congruent color (e.g., the word “green” written in green color), and follows with a list in incongruent color (e.g., the word “green” written in red color). It is well established that processing the content of the word is more automatic than processing the color of the word. Therefore, in the incongruent condition, the individual needs to inhibit the response of word naming that competes with the response of color naming. In our experimental MEG set-up, words were presented to participants via a video projector, and the children’s responses were monitored on-line to check for errors. Besides the congruent (termed “Congruent ink” in this study) and incongruent conditions (“Incongruent ink”), we also conducted a baseline condition (“Black-ink”) in which participants named the color words written in black ink, where interference effects were expected to be much lower or absent. Ninety four words were presented for each condition (Black-ink, Congruent-ink, and Incongruent-ink), the time interval between words was 2.5 s.

### Auditory attention task

The auditory task included two conditions with varying attentional requirements. In the simple reaction to stimulus, that is, a “low attention” condition (which we term “No attend” in this work) the participants heard repeated identical auditory tones and were instructed to press the response button to every tone. In the auditory oddball condition (“Attend” condition), that required attention to a deviant tone amongst otherwise common tones, participants pressed the response button only after hearing a deviant tone. In this way, the “low-attention” condition mainly reflected sensory registration of auditory stimuli, and the oddball condition reflected decision-making based on an auditory distinction. The baseline recording for this task was a period of 30–60 s when individuals were asked to remain quiet and did not receive any auditory input. Tones were presented binaurally with a 750 ms inter-stimulus interval. MEG recording time was 5 min for the low-attention and oddball conditions. There were 400 of the same stimuli presented in the low-attention condition. There were equally 400 stimuli presented in the oddball condition, of which 30% were deviant tones.

## Phase synchronization analysis

Visual inspection of the MEG recordings for artifacts was done during the acquisition and off-line before the analysis to remove sensors with artifacts or repeat the acquisition. Recordings were initially band-passed using a FIRLCS filter with a band-pass of ±2 Hz around a “*central frequency*”. The band-pass filtering done before the extraction of the oscillation phase removes eye blink artifacts (which tend to appear in frontal sensors) because these last around 300–400 ms, which is ∼2.5–3.3 Hz in terms of frequencies. Since in our study the lowest frequency studied is 10 ± 2 Hz, we can consider that eye blinks are not affecting our results. In this study, we used four central frequencies, 10, 18, 26 and 32 Hz, thus covering the range 8–34 Hz. The reason to choose these frequency bands is that they cover most of the ranges from α to lower γ that have been attributed to cognitive task performances. In addition, due to some limitations with the extraction of the phase using the Hilbert transform, especially the advice to have about 20 points per characteristic period of the oscillation (see page 367 in Pikovsky et al., 2001), phase synchrony was not assessed past 34 Hz.

On these band-passed signals, the Hilbert transform was applied and successive values of instantaneous phases were derived from the corresponding analytic signal. These phase series were then analyzed using sliding windows extracting the Mean Phase Coherence Statistic between two MEG recording channels as described in Mormann et al. (2000). Briefly, we use the analytic signal approach, employing the Hilbert transform to estimate instantaneous phases and calculate phase locking between two MEG recording channels (sensors), as previously described (Garcia Dominguez et al., 2005, 2007). With noisy data, phase synchronization has to be defined in a statistical sense: two signals are phase synchronized if the difference between their phases is bounded over a selected time window, that is, if it clusters around a single value (Pikovsky et al., 2001). A measure of this is the circular variance (CV) of the phase differences Δ*θ*(*t*), or alternatively, the coefficient *R* = 1 CV, which can also be expressed as:

$$R_{jk}=\left|<\exp\;(\;i\Delta \theta_{jk}(t)\;)>\right|$$

Here |·| denotes the norm and <·> the mean value. $\Delta \theta_{jk}(t)=\theta_{j}(t)-\theta_{k}(t)$ are the series of phase differences between the analytic signals of series indexed by *j* and *k* (each index *j* and *k* refer to one signal, that is, one MEG sensor time series) over a given time window. The value of *R* varies from 0 to 1, the higher the value the tighter the clustering of the phase differences Δ*θ* about a single mean value; that is, the closer the *R*-value to 1 the more synchronized the signals.

To estimate the mean synchrony index in the Stroop task, as described in detail in Pérez Velázquez et al. (2009), averages of the values of the synchronization index *R* were computed from stimulus presentation to the moment near the individual’s response, about 0.45–0.6 s after stimulus presentation in the Stroop task. The precise time to calculate the average varied slightly from individual to individual because the time to answer was variable and the average of the synchrony index was taken from the time of stimulus presentation to just before the subject’s response. For this purpose, the minimum time for each response of the individual rather than the mean of each subject’s distribution of reaction times was taken. All 282 trials (94 words × 3 conditions) were used for the analysis. The “baseline” was the initial list of words written in black ink. The results derived from the estimations of the magnitude of synchrony in this task were reported in Pérez Velázquez et al. (2009). In the present study, those synchronization indices estimated in the previous study were used to construct the edf to be analyzed as described below.

For the auditory attention task data, synchrony between two cortical sensor groups (those aforementioned above and shown in Figure 1) was computed, as in the Stroop task, using the average of all sensor combinations between the regions. For example, we selected 6 left parietal sensors and 6 right temporal sensors and formed 36 inter-group pairs. For each task condition, the synchrony values between these 36 sensor pairs were averaged to define the average synchrony index between the two sensor groups at each time point. Unlike in the Stroop task, in this case the synchrony index was not calculated phase-locked to the stimulus presentation, rather was calculated in a sliding window of 1 s for the whole 5 m recording (this is reasonable as attention, in this task, is supposed to be continuous and not intermittent). This derived average was then compared between subject groups and between conditions, and was used to obtain the empirical distribution functions.

## Testing power law distribution

We used the method described in Janczura and Weron (2012), which is based on the asymptotic properties of the edf. Details and validation of the procedure can be found in that article. We used the edf (the sample estimate of the cumulative distribution function) of the phase synchronization index rather than probability densities (pdf) because the former is not as biased as the pdf in terms of binning the data points that is required to construct a pdf but not an edf, and in general tests on edf are more powerful than those done on pdf (Newman, 2005). Especially, it has been documented that the cdf is more accurate to fit power laws (Dehgani et al., 2012). To construct the edf, the values of the *R* index were not further averaged in the Stroop task because the values represented the average from stimulus presentation to the moment near the individual’s response in the time periods mentioned in the previous paragraph on phase synchrony analysis. In the auditory task, the *R*-values, computed in a sliding window as mentioned above, were averaged in 1-s windows to reduce the number of data points (otherwise we would have 625 points per second, as we used a 625 Hz acquisition rate, that would result in a very large number of data points for the 5 m MEG time series and for all sensor combinations) and to make it more comparable to the Stroop task data. In total and for each individual and each task, the number of data points (that is, the *R*-values corresponding to the sensor combinations) used to derive the edf was 864 in the Stroop task, 4437 in the attention task, and 387 in the “baseline” for the attention task (because here the recordings were of shorter duration).

**Figure 2:**
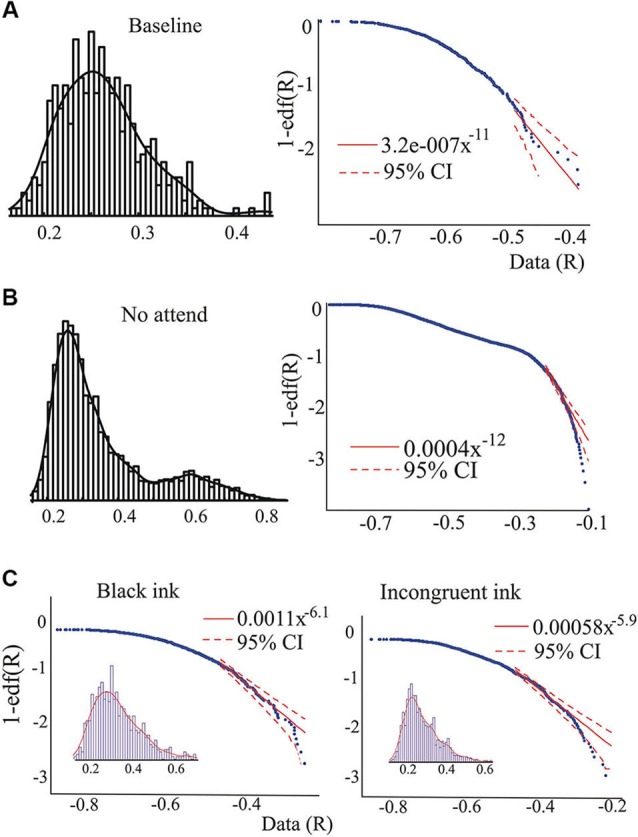
**Examples of the pdf and its characteristics**. **(A)** and **(B)**, pdf of the synchrony index and the edf corresponding to the baseline and the “not attend” conditions of one individual (non-ASD) performing the auditory attention task. In the right-hand side graphs, the logarithmic plots of the edf versus the data (*R*-values) are presented. Dashed red lines indicate 95% confidence for a power law, showing the presence of outliers in **B**. Note as well the change in the pdf, becoming almost bimodal in **B**, and as well the possible presence of a power law regime in the middle of the edf. Fitting the power law in this middle range (70–30% of the values, see Section Materials and Methods for ranges used), the exponent found was 2.4. **(C)** Data collected from another individual (non-ASD) during performance of the Stroop task, showing the presence of outliers in the tail during the incongruent color condition.

**Figure 3:**
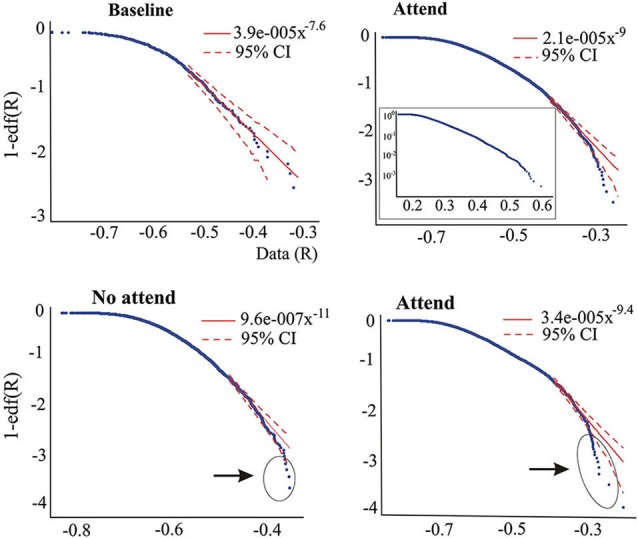
**Upper graphs correspond to one subject (ASD) performing the auditory attention task, illustrating that the tail power law characteristics disappear during the attend condition**. The inset on the right-hand side graph is the log-linear plot, suggesting that the edf has more pronounced exponential characteristics rather than power law features. Lower plots are from another subject (non-ASD) showing the presence of outliers in both conditions of the auditory task, but more numerous in the “attend” condition (circled). As in Figure 2B; the middle part (70–40%) of the edf in the “Attend” condition could be fit to a power law with exponent 3.4.

In brief, Janczura and Weron’s MATLAB algorithm (CI_powertail.m) estimates confidence intervals of a specified significance level (set at 0.05 in this study) for a power law fitted to a certain range of the edf. The logarithmic plots (Figures 2 and 3) represent 1-edf on the *y*-axis and the data on the *x*-axis. The ranges used in our study to fit the tail power law were (unless otherwise stated in the text) to the largest 5–1% values for the “attend” and “no attend” conditions of the auditory task, and 25–2.5% in the baseline condition of the auditory task and for the Stroop task. The ranges had to differ because of the different number of data points as detailed above. When the power law was fitted to central regions of the edf, the range was 70–25%.

To assess possible phenomenological bifurcations (Kuehn, 2011), we estimated whether two pdfs of the *R* indices were statistically different using the two-sample Kolmogorov-Smirnov test, with the null hypothesis that the two data sets are from the same continuous distribution.

## Results

In order to inspect characteristics of the phase synchrony probability distribution function or the edf, a computation of the phase synchrony index, described in Section Materials and Methods, was done first. It should be noted, as discussed below in the Discussion section, that our synchrony analysis among MEG sensors reflects population-scale levels of activity in large cellular ensembles, mostly a combination of synaptic potentials and neuronal action potentials (Toga and Mazziotta, 2002), thus the synchrony index in reality represents correlated phases among the MEG sensors. The average magnitude of the synchrony revealed few differences between the ASD and the control (non-ASD) group during task performance. The most notable difference is that the slight increase in synchrony during performance of the auditory attention task was more evident in the non-ASD group, as presented in Figure 1 for the central frequency of 10 Hz. Note that the synchrony index between two sensor groups tends to augment from the “control”, or baseline, to the “attend” condition in both ASD and non-ASD participants, and that it is already higher than baseline in the “no-attend” condition. Apparently, only the fact that participants had to perform a task either paying or not paying attention to deviant tones as instructed, already changes the brain synchrony patterns. In contrast, and shown as well in Figure 1, no apparent reproducible change in synchrony is detected when evaluated at 32 Hz (or at 26 Hz, not shown). The increase in the synchrony index *R* associated with task performance was observed when phase synchrony was computed at central frequencies of 10 and 18 Hz, and the relative changes during task performance, between the “baseline” and “attend” condition, were an increase of 13.1 ± 5.6% and 5.54 ± 4.6% for the non-ASD group at 10 and 18 Hz respectively, and 4.4 ± 3.5 and (a decrease of) −0.6 ± 1.9% for the ASD group. Thus it seems that it is around the α-frequency range (10 ± 2 Hz) where the tendency to enhance the magnitude of phase synchrony amongst the MEG sensors assessed is more pronounced. Note that, in the ASD group, the magnitude of synchrony at 32 Hz is highest in the parietal sensors (LP-RP) regardless of task condition, as indicated as well in previous studies (Pérez Velázquez et al., 2009; Teitelbaum et al., 2012). Comparison of the number of errors (deviant tones not detected) committed during the performance of the oddball task (“attend” condition) did not significantly differ between the two groups, although there was a trend for worse performance by those with ASD (ASD mean of 12.25 ± 13 errors; control group mean of 10.1 ± 7.3).

The changes in synchrony during the Stroop task were reported in Pérez Velázquez et al. (2009), so it will not be reproduced here. Briefly, significant increases in the magnitude of the synchrony index were observed in the non-ASD participants during the “incongruent” condition, but no apparent change in synchrony between conditions was detected in the ASD group. The behavioral results of this task are also reported in that paper: the difference in the errors committed (reading the word rather than naming the color in the incongruent condition) between the two groups of children was not significant even though, as found in the attention task, there was a tendency to make more mistakes in the case of ASD subjects (average of 5 ± 4.8 errors for the ASD participants versus 2.68 ± 2.5 errors in the control group).

Whereas the averaged magnitudes of synchronization provide certain information regarding brain dynamics, in addition to presenting the averages it is also informative to inspect the whole pdf of the magnitudes of synchrony, and this was our main purpose in the present study. As is well known, when pdfs are not Gaussian the central tendencies (median, most probable value, average) will differ, so which one to use is a matter of convenience or taste, thus inspecting characteristics of the whole pdf, especially the tails, provides a more complete picture than averages and variances alone. Figure 2 shows two pdfs of the synchronization index evaluated at 26 Hz for one subject performing the auditory task, Figure 2A is that derived from the baseline condition and Figure 2B for the “no-attend” condition. Note the differences in shape, one (“no-attend”) being bimodal, differences that can be quantified by a Kolmogorov-Smirnov (KS) test (in this case the difference is very significant: *p* < 0.0001). The logarithmic plot of the edf (or rather 1-edf, as noted in Section Materials and Methods) is represented in the same figure, and these were used to assess the presence of power law in the tails, as explained in Section Materials and Methods. Note that some power laws could be present not in the tails of the distribution but in the middle, as in Figure 2B (the straight segment in the middle), however this was uncommon (less than 45% of inspected edfs). Power laws in the tails were not too frequently found either.Tables 1 and 2 provide the abundance of power laws found in the tails: in average in the non-ASD group, these were present in 27.9% (auditory task) and 32.1% (Stroop task) of those evaluated, and in the ASD group the averages were 35% (auditory task) and 39.3% (Stroop). Other values are presented in the tables, where it can be seen a disappearance of tail power law characteristics with increasing task cognitive effort, an effect seen in both tasks. Figure 2C shows the loss of tail power law (appearance of outliers) in the “incongruent ink” condition of the Stroop task, the more demanding of the three in that task. Perhaps because of this effect, notice in Tables 1 and 2 that, for the “baseline” conditions in both tasks, the frequency of tail power laws is greater in the baseline condition for the auditory task (60.5% of instances), when children were asked to remain relaxed, whereas in the Stroop task (36.4% of instances) the cognitive load was higher as they had to read a list of words in black ink. Thus, less power law features are associated with more cognitive effort. The Discussion section contains comments on why the power law regime in the synchrony distribution is less frequent as cognitive load increases. Representative examples are presented in Figures 2 and 3. Even when power laws could not be fitted, there were more outliers as cognitive effort increased, depicted in Figure 3 (lower graphs) and quantified in Table 1 (“points out of PL”). It is worth noticing that rather than power laws, some of the edfs had exponential characteristics, as shown in Figure 3, upper graph inset.

**Table 1:** **Presence of tail power law (PL) regimes in the distribution of the synchronization index during the auditory attention task**.

		**Baseline**	**Not Attend**	**Attend**
Control group (*n* = 13)	Percentage PL	57.4%	12.5%	15.4%
	Points out of PL	4 ± 3	13.2 ± 9.7	14.5 ± 9
ASD group (*n* = 12)	Percentage PL	64.1%	20.8%	25.5%
	Points out of PL	2.1 ± 1.8	11.6 ± 7.8	10.6 ± 8.5
Total (*n* = 25)	Percentage PL	60.5%	16.7%	20.2%

Data points outliers (“Points out of PL”) are more numerous as the attentional demand increases, from baseline to the “Attend” condition (lower plots in Figure 3 depict one example).

**Table 2:** **Presence of tail power law (PL) regimes in the distribution of the synchronization index during the Stroop colour-word task**.

		**Black ink**	**Congruent ink**	**Incongruent ink**
Control group (*n* = 7)	Percentage PL	46.4%	25%	25%
ASD group (*n* = 7)	Percentage PL	25.9%	39.3%	32%
Total (*n* = 14)	Percentage PL	36.4%	32.1%	28.6%

The tendency to change in pdf characteristics (as represented in Figure 2) is suggestive of a critical transition, what is known as phenomenological bifurcations (Kuehn, 2011), that describe changes in the probability density functions in random dynamical systems. To quantitatively assess the difference between pdfs in each condition of the tasks, KS tests were used. Of 388 pdfs evaluated, including all children and all tasks (thus 388/2 = 194 transitions, one “transition” here means going from one task condition, say “congruent ink”, to the next, “incongruent ink”), changes between pdfs were significant (*p* < 0.05) 52.1% of times, but were more abundant when there was a transition from power-law to non-power law (or vice versa) characteristics (53.9% of the times) than when there was no such transition (39% of the times). This difference was more pronounced in the data corresponding to the Stroop task (47.2% of instances for power law to non-power law, and 25% for the other case) than in the auditory attention task. These observations suggest that a bifurcation, manifested as a change in the characteristics of the pdf, may take place when increasing cognitive load of a task.

## Discussion

Critical dynamics, the behavior of extended systems near a phase transition where scale invariance prevails, has been proposed for nervous system activity as it has several features that optimize information processing (Beggs, 2007; Shew and Plenz, 2013), and this notion has been taken with such enthusiasm that the field is currently in the grip of an explosion of fecundity. Indications of such dynamics have been reported for a variety of *in vitro* and *in vivo* recordings, ranging from *in vitro* slice electrophysiology to human functional magnetic resonance imaging. However, there still remains considerable debate as to whether brains really operate close to criticality rather than, for instance, stochastic or oscillatory dynamics. One sign of criticality that has become a favorite is the inspection of power-law distributions in nervous system data, and such power-law scaling has been reported associated with different types of recordings, even though some studies failed to find clear evidence. Here, we studied whether there is a power law scaling in the distribution of the phase synchronization derived from magnetoencephalographic recordings during executive function tasks performed by children with and without ASD. Our observations suggest that power law scaling of phase synchrony indices derived from MEG recordings is not very common in both ASD and non-ASD groups and its frequency of occurrence tends to diminish with increased cognitive load/effort as children performed the tasks. There were indications of changes in the phase synchrony probability distribution functions associated with a transition from power law scaling to lack of power law, perhaps suggesting the presence of phenomenological bifurcations in brain dynamics associated with cognitive load. Hence, the observations of power law and other (exponential) scaling regimes plus the signs of phenomenological bifurcations, further support the metastability of brain dynamics and suggest that some brain areas experience critical transitions.

Our studies are based on the calculation of a phase synchronization index from MEG recordings that reflect large-scale activity, at the collective level, in extensive cellular ensembles. The synchrony index thus represents correlated activity in brain areas over which the sensors locate. There are certain limitations worth noting. Perhaps principally, the signals detected may summate at nearby MEG sensors, depending on the intensity of the source, causing multiple sensors to contain similar activities. To minimize summation of signals, the areas of sensors chosen were not direct neighbors. These sensors were chosen as well because the cortical areas over which they are located are associated with sensorimotor transformations (Binkofski et al., 1999). Estimating the time series at the source level (in brain tissue) is a solution to overcome the summation at neighboring sensors, and while some methods to derive the signals at the sources have been reported in the literature, source reconstruction adds another level of complexity to the analysis and may even yield spurious results, as it is an “ill-posed mathematical problem” (Gross et al., 2013), mainly because assumptions must be made about the origin and location of the expected sources in order to properly constrain the solution to the problem, and thus there is bias as it is not trivial to choose what brain areas could be expected to account for the brain dynamics. With these considerations in mind, our analyses are performed at the sensor level and the conclusions we draw from them focus on relative changes without focusing on specific cortical areas.

Traditional scientific reporting methods normally use averages and variances, which tend to hide the variability and fluctuations in data sets. Thus, a complementary approach is the observation of the full pdf. As evidenced in the figures, power law scaling could always be found in some segments of the edf, a well-known feature as few real-world distributions follow a power law over the entire range (Newman, 2005). This imposes a certain arbitrary constraint, in that one must choose a range of values at which the power law may hold, choice that is not trivial when using empirical data, and thus the scaling exponent will vary depending on the chosen data points. It is known as well that two or more power law regimes with different exponent may be present in the same distribution. To make things more complicated, the exponent values depend on sampling and several other aspects (Priesemann et al., 2009; Touboul and Destexhe, 2010; Marković et al., 2013). For all these reasons, we do not emphasize the values of the exponents in our work, nevertheless we note that the values of the exponent, either in the tail or in the middle of the distribution are larger than 2 (see legends of Figures 2B and 3, where a power law approximated to the middle part of the edf provided exponents >2). Because the classical exponent associated with self-organized criticality is 1, the celebrated 1/f scaling (Bak et al., 1988; Pritchard, 1992), exponents larger than 1 may not be associated with this phenomenon. High values of exponents have been recently reported in recordings from cat, monkeys and humans (Touboul and Destexhe, 2010; Dehgani et al., 2012), hence the matter of self-organized criticality in nervous system activity remains unclear at the present time. Nevertheless our study is not intended to present evidence for self-organized critical dynamics in brain synchronization, rather to inspect certain properties of the distributions of our synchrony index associated with performance of executive function tasks in two groups of individuals. In instances where power law regimes co-exist with others (e.g., exponential) in the distributions of synchrony magnitudes, it could be hypothesized that this is a sign of the metastability of brain dynamics, a notion proposed by several authors (Bressler and Kelso, 2001; Fingelkurts and Fingelkurts, 2004; Kelso, 2008; Pérez Velázquez and Frantseva, 2011; Deco and Jirsa, 2012; Kelso et al., 2013). Incidentally, one of the first early proposals of the brain as “organ whose natural state is one of unstable equilibrium” is due to William James in his 1879 essay “Are we automata?” published in *Mind*, 4, 1–22.

One aspect that, in principle, could be concluded from our study is that the power law features become less frequent as tasks require more effort/cognitive component. Since we investigated the edf of a synchronization index amongst MEG sensors, and if we assume these indices represent correlated activity in brain areas over which the sensors are positioned as expounded above, power law scaling then denotes that synchrony has no characteristic scale, and the absence of power law indicates that there are characteristic scales in synchrony; in the case of the (right-hand side) tails of the distribution, the absence of power law features means that there appear some especially high values of the magnitude of synchrony, perhaps because of the change in coordinated activity in certain cortical areas associated with task performance. Thus, the possible reason why we observed decrease incidence of power law regimes as cognitive effort augments can be explained by the associated enhanced synchronization needed to perform the task. In fact, Figure 1 indicates a tendency to increase synchrony during task performance. Equally, in the Stroop task, it was previously reported (Figures 1 and 2 in Pérez Velázquez et al., 2009) an increase in the magnitude of phase synchrony going from the baseline (“black ink”) to the “incongruent” condition (that is, the most difficult of the three conditions in that task) only for the non-ASD group, and consequently, notice in Table 2 the reduced occurrence of tail power laws for this group as task difficulty increased, but not for the ASD set. If some specific cortical areas become more synchronous, this will result in high values of the magnitude of synchrony (our *R* index) and therefore the loss of scale-free features. Heavy tails have been associated with small world features (Feldt et al., 2011), that applied to our studies would suggest there are highly “connected” cortical regions whereas most have low connections. Or more accurately, because phase synchrony as evaluated here is not really a measure of connectivity but a correlation between phases of oscillations, those results could be interpreted as few regions with highly correlated phases of the oscillations (cautionary notes on the notion of “connectivity” derived from these types of analyses have been presented elsewhere, Perez Velazquez, 2012). It is of interest that, in experiments *in vitro*, enhancing excitation using blockers of GABAergic transmission results in deviations from the neuronal avalanche power law observed in unperturbed brain slices (Beggs and Plenz, 2003). It is conceivable that this *in vitro* manipulation shares similar neurophysiological features with increasing cognitive effort, perhaps increased activity/excitation in some cortical regions, and therefore both results, *in vitro* and ours *in vivo*, are complementary.

In our study we have not emphasized the possible association of the observed power law regimes and self-organized criticality, because, as noted above, it is still inconclusive that power law scaling is directly related to self-organized criticality in nervous systems. Indeed, features of critical dynamics emerge in various situations even when the dynamics are not critical, as shown in networks that possess a hierarchical modular structure (Friedman and Landsberg, 2013) or a noisy feedforward structure (Benayoun et al., 2010). While indications of criticality derived from “neuronal avalanches” of activity (Beggs and Plenz, 2003) or the scaling of fluctuations in functional brain imaging (Fraiman and Chialvo, 2012) have been reported, other studies have cast some doubt as to the methods used to assess power laws in brain recordings (Clauset et al., 2009; Dehgani et al., 2012). For instance, Touboul and Destexhe (2010) observed that sometimes the scaling behavior is a consequence of the thresholding method, which applies to amplitude-based recordings. There is doubt too as to the generic character of this presumed criticality in nervous tissue (Bédard et al., 2006; Beggs and Timme, 2012). To complicate matters, power laws can be generated in a variety of manners (Reed and Hughes, 2002; Marković and Gros, 2014). Nevertheless, our finding of some signs of phenomenological bifurcations most commonly associated with transitions from power law to non-power law regimes, may suggest that, in some instances, our MEG recordings display signatures of possible phase transitions and thus provides a, perhaps indirect, support for criticality in some instances. The observation that power law regimes are not frequently seen may present another indication of criticality, because in principle it is only at the bifurcation point when power laws should be apparent, but once the transition has taken place, other regimes can be present; this is an important point, many times overlooked, mentioned in Beggs and Timme (2012). To stress it again, what has been demonstrated beyond doubt is that in systems at thermodynamic equilibrium power laws are found only near bifurcations, but in far from equilibrium conditions, this remains unclear. In view of what we, and others, have been reporting with regards to the apparent mixture of regimes, especially exponential (which is related to Poisson-type stochastic processes) and power-law scaling, brain recordings may represent the activity of coupled oscillator phenomena (Perez Velazquez et al., unpublished observations) in stochastic settings (Teramae and Tanaka, 2004; section 1.5 in Pérez Velázquez and Frantseva, 2011). For instance, Reed and Hughes (2002) reported that randomly observed stochastic processes exhibit tail power laws, and Deco and Jirsa (2012) proposed that resting state networks in the brain emerge as structured noise fluctuations in a multistable attractor landscape.

In terms of synchronization in the brain, the presence or absence of characteristic scales makes sense according to what has been found regarding, for instance, the stability of certain functional nets derived from EEG recordings (Chu et al., 2012), phenomenon which would require characteristic scales if we assume those stable nets are almost always functionally “connected”, whereas scale invariance makes sense too as many brain nets have to be loosely or very transiently coordinated, and especially when analyzing such recordings like MEG or EEG representing global, collective activities in myriad of cells. These neurophysiological features would support the varied dynamic behaviors of brain networks and in general metastable dynamics.

We have used phase synchronization in this study to evaluate power law scaling, instead of others most commonly used such as the size of bursts or number of spikes in neuronal avalanches. It is difficult to ascertain what type of metric is the best suited to characterize collective brain dynamics, but synchronization has two advantages. First, it seems to be a reasonable metric to scrutinize collective network dynamics, and it is and has been very widely used to study cognition and brain pathologies. The second advantage over other metrics that have been used in this type of studies is that a threshold is not needed to define the characteristic to be analyzed (it was mentioned above the problem with threshold-based methods to assess power law regimes, Touboul and Destexhe, 2010). Using different metrics to scrutinize for criticality will be crucial in the future, considering the controversies with the study of neuronal avalanches.

To conclude, a few comments on what these results may indicate about ASD brain dynamics. It was noted in the Introduction section the current debate about the classical notion of “underconnectivity” in view of recent observations suggesting, if something, the opposite. Since the time when specific changes in brain dynamics were proposed to account for ASD cognitive features, including the temporal binding deficit (Brock et al., 2002) and disruptions of coordinated timing in cellular activity and associated synchronization dynamics (Herbert, 2005; Uhlhaas and Singer, 2007), many reports have appeared indicating, sometimes, contrasting evidence. This should not be surprising if we consider the wide spectrum of autistic syndromes, and of course the great variety in the experimental and analytical methods used to assess brain dynamics. In our study, no main differences were found comparing the ASD and the non-ASD participants, other than a tendency to exhibit more synchrony in non-ASD individuals when performing the tasks, thus having in general less frequent power law features than in the ASD data (see percentages in the tables). Thus, the current assortment of observations seems to indicate that, as we already noted in previous publications (Pérez Velázquez and Frantseva, 2011; Garcia Domínguez et al., 2013; Pérez Velázquez and Fernández Galán, 2013), it may not be a matter of more or less connectivity in the ASD brain, rather a different type of brain coordinated activity that manifests in the particular information processing characteristics and associated special cognitive style of individuals with autism.

## Conflict of interest statement

The authors declare that the research was conducted in the absence of any commercial or financial relationships that could be construed as a potential conflict of interest.

## Footnotes

^1^see for instance http://sfari.org/news-and-opinion/news/2013/autism-brains-are-overly-connected-studies-find for recent perspectives on the topic

## References

[B1] Bak P. Tang C. Wiesenfeld K. (1988). Self-organized criticality. Phys. Rev. A 38, 364–374 10.1103/PhysRevA.38.364 9900174

[B2] Bédard C. Kröger H. Destexhe A. (2006). Does the 1/f frequency scaling of brain signals reflect self-organized critical states? Phys. Rev. Lett. 97:118102 10.1103/physrevlett.97.118102 17025932

[B3] Beggs J. M. (2007). The criticality hypothesis: how local cortical networks might optimize information processing. Philos. Trans. A Math. Phys. Eng. Sci. 366, 329–343 10.1098/rsta.2007.2092 17673410

[B4] Beggs J. M. Plenz D. (2003). Neuronal avalanches in neocortical circuits. J. Neurosci. 23, 11167–11177 14657176 10.1523/JNEUROSCI.23-35-11167.2003 PMC6741045

[B5] Beggs J. M. Timme N. (2012). Being critical of criticality in the brain. Front. Physiol. 3:163 10.3389/fphys.2012.00163 22701101 PMC3369250

[B6] Benayoun M. Cowan J. D. van Drongelen W. Wallace E. (2010). Avalanches in a stochastic model of spiking neurons. PLoS Comput. Biol. 6:e1000846 10.1371/journal.pcbi.1000846 20628615 PMC2900286

[B7] Binkofski F. Buccino G. Posse S. Seitz R. J. Rizzolatti G. Freund H. (1999). A fronto-parietal circuit for object manipulation in man: evidence from an fMRI study. Eur. J. Neurosci. 11, 3276–3286 10.1046/j.1460-9568.1999.00753.x 10510191

[B8] Bressler S. L. Kelso J. A. (2001). Cortical coordination dynamics and cognition. Trends Cogn. Sci. 5, 26–36 10.1016/s1364-6613(00)01564-3 11164733

[B9] Brock J. Brown C. C. Boucher J. Rippon G. (2002). The temporal binding deficit hypothesis of autism. Dev. Psychopathol. 14, 209–224 10.1017/s0954579402002018 12030688

[B10] Chialvo D. R. (2010). Emergent complex neural dynamics. Nat. Phys. 6, 744–750 10.1038/nphys1803

[B11] Christensen K. Moloney N. R. (2005). Complexity and Criticality. London: Imperial College Press

[B12] Chu C. J. Kramer M. A. Pathmanathan J. Bianchi M. T. Westover M. B. Wizon L. (2012). Emergence of stable functional networks in long-term human electroencephalography. J. Neurosci. 32, 2703–2713 10.1523/jneurosci.5669-11.2012 22357854 PMC3361717

[B13] Clauset A. Shalizi C. R. Newman M. E. J. (2009). Power-law distributions in empirical data. SIAM Rev. 51, 661–703 10.1137/070710111

[B14] Deco G. Jirsa V. K. (2012). Ongoing cortical activity at rest: criticality, multistability, and ghost attractors. J. Neurosci. 32, 3366–3375 10.1523/jneurosci.2523-11.2012 22399758 PMC6621046

[B15] Dehgani N. Hatsopoulos N. G. Haga Z. D. Parker R. A. Greger B. Halgren E. (2012). Avalanche analysis from multielectrode ensemble recordings in cat, monkey and human cerebral cortex during wakefulness and sleep. Front. Physiol. 3:302 10.3389/fphys.2012.00302 22934053 PMC3429073

[B16] Feldt S. Bonifazi P. Cossart R. (2011). Dissecting functional connectivity of neuronal microcircuits: experimental and theoretical insights. Trends Neurosci. 34, 225–236 10.1016/j.tins.2011.02.007 21459463

[B17] Fingelkurts A. A. Fingelkurts A. A. (2004). Making complexity simpler: multivariability and metastability in the brain. Int. J. Neurosci. 114, 843–862 10.1080/00207450490450046 15204050

[B18] Flohr H. (1995). Sensations and brain processes. Behav. Brain Res. 71, 157–161 10.1016/0166-4328(95)00033-X 8747183

[B19] Fraiman D. Chialvo D. R. (2012). What kind of noise is brain noise: anomalous scaling behavior of the resting brain activity fluctuations. Front. Physiol. 3:307 10.3389/fphys.2012.00307 22934058 PMC3429078

[B20] Friedman E. J. Landsberg A. S. (2013). Hierarchical networks, power laws and neuronal avalanches. Chaos 23:013135 10.1063/1.4793782 23556972 PMC3606226

[B21] Frith U. (1989). Autism Explaining the Enigma. Oxford: Blackwell Publishing

[B22] Garcia Domínguez L. Pérez Velázquez J. L. Fernández Galán R. (2013). A model of functional brain connectivity and background noise as a biomarker for cognitive phenotypes: application to autism. PLoS One 8:e61493 10.1371/journal.pone.0061493 23613864 PMC3629229

[B23] Garcia Dominguez L. Wennberg R. Gaetz W. Cheyne D. Snead O. C. Perez Velazquez J. L. (2005). Enhanced synchrony in epileptiform activity? Local versus distant phase synchronization in generalized seizures. J. Neurosci. 25, 8077–8084 10.1523/jneurosci.1046-05.2005 16135765 PMC6725453

[B24] Garcia Dominguez L. Wennberg R. Perez Velazquez J. L. Guevara Erra R. (2007). Enhanced measured synchronization of unsynchronized sources: inspecting the physiological significance of synchronization analysis of whole brain electrophysiological recordings. Int. J. Phys. Sci. 2, 305–317 10.1186/1471-2202-8-s2-p41

[B25] Georgelin Y. Poupard L. Sartène R. Wallet J. C. (1999). Experimental evidence for a power law in electroencephalographic α-wave dynamics. Eur. Phys. J. B 12, 303–307 10.1007/s100510051008

[B26] Gross J. Baillet S. Barnes G. R. Henson R. N. Hillebrand A. Jensen O. (2013). Good practice for conducting and reporting MEG research. Neuroimage 65, 349–363 10.1016/j.neuroimage.2012.10.001 23046981 PMC3925794

[B27] Herbert M. R. (2005). Large brains in autism: the challenge of pervasive abnormality. Neuroscientist 11, 417–440 10.1177/0091270005278866 16151044

[B28] Janczura J. Weron R. (2012). Black swans or dragon-kings? A simple test for deviations from the power law. Eur. Phys. J. Spec. Top. 205, 79–93 10.1140/epjst/e2012-01563-9

[B29] Kanner L. (1943). Autistic disturbances of affective contact. Nerv. Child 2,217–250 4880460

[B30] Kelso J. A. (2008). An essay on understanding the mind. Ecol. Psychol. 20, 180–208 10.1080/10407410801949297 19865611 PMC2768408

[B31] Kelso J. A. Dumas G. Tognoli E. (2013). Outline of a general theory of behavior and brain coordination. Neural Netw. 37, 120–131 10.1016/j.neunet.2012.09.003 23084845 PMC3914303

[B32] Kuehn C. (2011). A mathematical framework for critical transitions: bifurcations, fast-slow systems and stochastic dynamics. Physica D 240, 1020–1035 10.1016/j.physd.2011.02.012

[B33] Langton C. G. (1990). Computation at the edge of chaos, phase transitions and emergent computation. Physica D 42, 12–37 10.1016/0167-2789(90)90064-v

[B34] Marković D. Gros C. (2014). Power laws and self-organised criticality in theory and nature. Phys. Rep. 536, 41–74 10.1016/j.physrep.2013.11.002

[B35] Marković D. Gros C. Schuelein A. (2013). Criticality in conserved dynamical systems: experimental observation vs. exact properties. Chaos 23:013106 10.1063/1.4773003 23556943

[B36] Mitzenmacher M. (2004). A brief history of generative models for power law and log-normal distributions. Internet Math. 1, 226–251 10.1080/15427951.2004.10129088

[B37] Mormann F. Lehnertz K. David P. Elger C. E. (2000). Mean phase coherence as a measure for phase synchronization and its application to the EEG of epilepsy patients. Physica D 144, 358–369 10.1016/s0167-2789(00)00087-7

[B38] Newman M. E. J. (2005). Power laws, Pareto distributions and Zipf’s law. Contemp. Phys. 46, 323–351 10.1080/00107510500052444

[B39] Packard N. (1988). “Adaptation towards the edge of chaos,” in Dynamic Patterns in Complex Systems, eds Kelso J. A. S. Mandell A. J. Shlesinger M. F. (Singapore: World Scientific), 293–301

[B40] Pérez Velázquez J. L. Fernández Galán R. (2013). Information gain in the brain’s resting state: a new perspective on autism. Front. Neuroinform. 7:37 10.3389/fninf.2013.00037 24399963 PMC3870924

[B41] Perez Velazquez J. L. (2012). The biophysical bases of will-less behaviours. Front. Integr. Neurosci. 6:98 10.3389/fnint.2012.00098 23109920 PMC3478585

[B42] Pérez Velázquez J. L. Barceló F. Hung Y. Leshchenko Y. Nenadovic V. Belkas J. (2009). Decreased brain coordinated activity in autism spectrum disorders during executive tasks: reduced long-range synchronization in the fronto-parietal networks. Int. J. Psychophysiol. 73, 341–349 10.1016/j.ijpsycho.2009.05.009 19465065

[B43] Pérez Velázquez J. L. Frantseva M. V. (2011). The Brain-Behavior Continuum—The Subtle Transition Between Sanity and Insanity. Singapore: World Scientific Publishing

[B44] Pikovsky A. Rosenblum M. Kurths J. (2001). Synchronization. Cambridge: Cambridge University Press

[B45] Priesemann V. Munk M. H. J. Wibral M. (2009). Subsampling effects in neuronal avalanche distributions recorded in vivo. BMC Neurosci. 10:40 10.1186/1471-2202-10-40 19400967 PMC2697147

[B46] Pritchard W. S. (1992). The brain in fractal time: 1/f-like power spectrum scaling of the human electroencephalogram. Int. J. Neurosci. 66, 119–129 10.3109/00207459208999796 1304564

[B47] Reed W. J. Hughes B. D. (2002). From gene families and genera to incomes and internet file sizes: why power laws are so common in nature. Phys. Rev. E 66:067103 10.1103/physreve.66.067103 12513446

[B48] Shew W. L. Plenz D. (2013). The functional benefits of criticality in the cortex. Neuroscientist 19, 88–100 10.1177/1073858412445487 22627091

[B49] Sornette D. (2004). Critical Phenomena in Natural Sciences. Berlin: Springer Verlag

[B50] Stroop J. R. (1935). Studies of interference in serial verbal reactions. J. Exp. Psychol. 18, 643–662 10.1037/h0054651

[B51] Teitelbaum A. Belkas J. Brian J. Perez Velazquez J. L. (2012). “Distinct patterns of cortical coordinated activity in autism,” in Autism Spectrum Disorders: New Research, eds Richardson C. E. Wood R. A. (Hauppauge, New York: Nova Science Publishers), 95–112

[B52] Teramae J. N. Tanaka D. (2004). Robustness of the noise-induced phase synchronization in a general class of limit cycle oscillators. Phys. Rev. Lett. 93:204103 10.1103/physrevlett.93.204103 15600929

[B53] Toga A. W. Mazziotta J. C. (2002). Brain Mapping—The Methods. Amstredam: Academic Press

[B54] Touboul J. Destexhe A. (2010). Can power-law scaling and neuronal avalanches arise from stochastic dynamics? PLoS One 5:e8982 10.1371/journal.pone.0008982 20161798 PMC2820096

[B55] Uhlhaas P. J. Singer W. (2007). What do disturbances in neural synchrony tell us about autism? Biol. Psychiatry 62, 190–191 10.1016/j.biopsych.2007.05.023 17631116

[B56] von der Malsburg C. (1981). The Correlation Theory of Brain Function. Göttingen: Max-Planck Institute for Biophysical Chemistry

